# Syphilis-Corpuscles (Selected)

**Published:** 1872-03

**Authors:** 


### SYPHILIS-CORPUSCLES.

Under date of Vienna, February 9, 1872, Dr. J. R. Chadwick writes to the Boston Medical and Surgical Journal:

Messrs. Editors,—Every chair and every square inch of standing room were filled this evening at the meeting of the "Gesellschaft Aertze," because of the advertised review of Dr. Lostorfer's discovery, by Prof. Wedl. Prof. Hebra in the chair.

Prof. Wedl, having first recounted the results of the examinations of Dr. Lostorfer, and the steps by which they were said to have been reached, stated that he had pursued the same method in his researches, but had failed to discover any corpuscles in the blood of syphilitics which were not to be found equally in the blood of healthy individuals. He has easily found bodies, similar to those described by Dr. Lostorfer as peculiar to syphilitic blood, in almost every specimen examined, both healthy and diseased. They were round, shining bodies, of less size than the blood corpuscles, and having a slightly bluish-green shimmer. Prof. Gruber (aurist) and Docent Dr. Neumann (dermatologist) saw these specimens, and pronounced them to be precisely similar to the bodies which they had seen in Dr. Lostorfer's preparations. Prof. Wedl had then made a preparation of mistura oleosa, in which the fat drops were finely broken up, and in which, on comparison with the so-called syphilis-corpuscles, the small fat drops could not be distinguished from the corpuscles. This applied only to the perfectly symmetrical round bodies. Besides these, bodies had been described of various forms, and in many cases supplied with one or more sprouts; these the Professor asserted to be "torn-off pieces of protoplasm." The formation of vacuoles was nothing more than a process of decomposition, in which carbonate of ammonia was formed, and which could be demonstrated any day by adding an alkali to fat, and watching their development in the fat. As to the supposed growth of the corpuscles, this the Professor asserted had not been proved, because Dr. Lostorfer had not shown that he had followed one and the same through its various stages of development; moreover, that he himself had watched these same bodies for several weeks, and failed to note the slightest increase in size. In order to remove the blood corpuscles, which had obscured the bodies in dispute, a stream of distilled water had been passed over the slide, and by this means the corpuscles left alone under the microscope. In conclusion, he suggested that the fat had been introduced into the specimen by accident, as, for instance, in pricking the finger to obtain a drop of blood, a few drops of fat might be easily squeezed from out the sebaceous follicles of the skin, and be brought upon the slide with the blood.

Prof. Stricker then rose to combat the scepticism of Prof. Wedl, with a reiteration of the extreme caution used in the tests to which Dr. Lostorfer had been subjected, and denied that the Professor could have seen the corpuscles in question—adding, further, that he himself had examined them many hundred times, and while unable to judge of their true nature, was convinced that they were neither molecules of fat nor protoplasmic bodies. Dr. Lostorfer inferred, that if, as asserted by Prof. Wedl, the bodies had been observed on the first day after the blood was drawn, they could not have been those described by him, which never had appeared within the first twenty-four hours. He further concluded, that if water had been added to the preparations, his corpuscles must have been destroyed, because he had proved them to be shriveled up on the admixture of water, especially in the earlier stages of their existence.

Prof. Stricker thought it likely that Prof. Wedl, by his stream of water, had at all events washed away the corpuscles together with the blood corpuscles, and had probably by its medium brought many new microscopic objects into the field.

Prof. Wedl replied that the "wet chamber" in which the slides were placed would in any case admit water to the preparation by the well-known principle of the diffusion of fluids, and that the streams thereby produced would effect the same as his method of washing the blood. He also thought that after twenty-four hours the blood corpuscles would have lost their color, and might be taken for strange bodies.

Dr. Neumann recounted some of Hallier's discoveries of spores indicative of various diseases, and his own experiments with the specimens supplied him by Hallier. The foreign and diagnostic bodies he had fully proved to be nothing but decomposed blood corpuscles. He had recently subjected syphilitic blood to constant observation for ten hours a day, and had invariably found the same appearances as in his previous researches.

Dr. Geber, the newly-appointed first assistant of Hebra, had, at the request of the latter, examined a large number of patients in the small-pox wards, and found, with the absence of syphilitic symptoms, many of these corpuscles, in part precisely similar to those shown him by Lostorfer, and in part of a somewhat more bluish-green tint. Moreover, in a dead subject, he had found them in great quantities.

Prof. Gruber testified to the exact resemblance of Prof. Wedl's specimens to those of Dr. Lostorfer.

It was finally decided that the matter of testing the possibility of a diagnosis of syphilis by means of these corpuscles should be referred to a committee, and the chair appointed the following members of the Society, omitting, in accordance with the wishes of those present, all who had taken any part in the discussion of the evening: Prof. Rokitansky (chairman), Profs. Brücke, Billroth, Reichert, Klob, Drs. Auspitz and Basche. To these was added the name of Prof. Karsten, after much excitement and opposition on the part of some present.

